# Perioperative predictors of outcome of hepatectomy for HBV-related hepatocellular carcinoma

**DOI:** 10.3389/fonc.2023.1230164

**Published:** 2023-07-13

**Authors:** Ziming He, Di Tang

**Affiliations:** Department of General Surgery, The Seventh Affiliated Hospital, Sun Yat-sen University, Shenzhen, China

**Keywords:** hepatitis B virus, hepatocellular carcinoma, predictor, preoperative, postoperative

## Abstract

Hepatitis B virus (HBV) is identified as a major risk factor for hepatocellular carcinoma (HCC), resulting in so-called hepatitis B virus-related hepatocellular carcinoma (HBV-related HCC). Hepatectomy for HCC is acknowledged as an efficient treatment strategy, especially for early HCC. Furthermore, patients with advanced HCC can still obtain survival benefits through surgical treatment combined with neoadjuvant therapy, adjuvant therapy, transcatheter arterial chemoembolization, and radiofrequency ablation. Therefore, preoperative and postoperative predictors of HBV-related HCC have crucial indicative functions for the follow-up treatment of patients with feasible hepatectomy. This review covers a variety of research results on preoperative and postoperative predictors of hepatectomy for HBV-related HCC over the past decade and in previous landmark studies. The relevant contents of Hepatitis C virus-related HCC, non-HBV non-HCV HCC, and the artificial intelligence application in this field are briefly addressed in the extended content. Through the integration of this review, a large number of preoperative and postoperative factors can predict the prognosis of HBV-related HCC, while most of the predictors have no standardized thresholds. According to the characteristics, detection methods, and application of predictors, the predictors can be divided into the following categories: 1. serological and hematological predictors, 2. genetic, pathological predictors, 3. imaging predictors, 4. other predictors, 5. analysis models and indexes. Similar results appear in HCV-related HCC, non-HBV non-HCV HCC. Predictions based on AI and big biological data are actively being applied. A reasonable prediction model should be established based on the economic, health, and other levels in specific countries and regions.

## 1 Introduction

**Figure 1:**
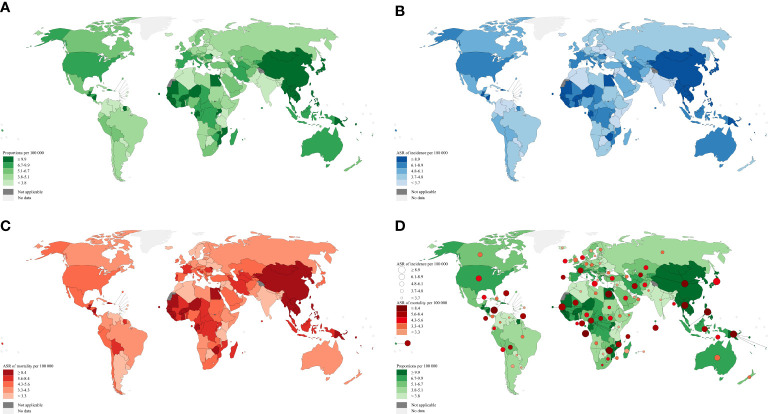
Global distribution map of liver cancer adapted from data source: GLOBOCAN 2020. Map production: IARC (http://gco.iarc.fr/today) World Health Organization. **(A)** Estimated number of prevalent cases (5-year) as a proportion in 2020. **(B)** Estimated age-standardized incidence rates in 2020. **(C)** Estimated age-standardized mortality rates in 2020. **(D)** Base map **(A)** + Size of the circle **(B)** + The color depth of circle **(C)**.

Primary liver cancer, in 185 countries and regions worldwide in 2020, is the sixth most commonly diagnosed cancer and the third leading cause of cancer death, with about 905,677 new cases and 830,180 deaths (**Figure 1**), 75%-85% of which are hepatocellular carcinoma (HCC) (1). As the main risk factor for HCC, a series of integrated global health sector strategies on hepatitis B virus (HBV) have been established at the 75th World Health Assembly, achieving global health impact with a significant reduction of new infections and mortality for the period 2022-2030 (2). HBV-related HCC refers to HCC caused by HBV, the main high-incidence area of which is the Asia-Pacific region, especially in China (3, 4). The pathogenesis is generally from the beginning of HBV infection, and then the occurrence of chronic hepatitis B (CHB), fibrosis, and cirrhosis, eventually leading to HCC. After HBV infection, the clinically common serum biomarkers include hepatitis B surface antigen (HBsAg), hepatitis B surface antibody, hepatitis B e antigen (HBeAg), hepatitis B e antibody, hepatitis B core antibody, and HBV DNA. As a new HBV infection biomarker, hepatitis B core-related antigen can still be detectable when HBV DNA level is undetectable or HBsAg is lost, because it is closely related to the presence of covalently closed circular DNA (cccDNA) in hepatocytes (5, 6). Hepatectomy is the main treatment strategy for early HCC, and even for advanced HCC combined with neoadjuvant therapy, adjuvant therapy, transcatheter arterial chemoembolization (TACE), and radiofrequency ablation (RFA). Besides the above biological indicators, numerous studies have focused on various preoperative and postoperative predictors to predict the prognosis of patients with feasible hepatectomy. This review summarizes various research results on preoperative and postoperative predictors of hepatectomy for HBV-related HCC over the past decade and previous landmark studies, including serology, molecular genomics, pathology, imaging, and other predictors (**Tables 1**–**3**), which are different from traditional factors of tumor status or liver function with the expectation of establishing correct disease prediction and guiding follow-up precise treatment.

**Table 1:** Predictors of worse prognoses.

Predictor	Classification	Related prognosis	Reference COI/threshold/value	Notes
AFP	*A, E*	OS, DFS, ER	400 ng/mL, 20 ng/dL, 529.8 ng/mL	a. Tumor marker for HCC b. Dynamic changes pre- and post-operatively a. Low specificity for cancer diagnosis
DCP	*A*	OS, DFS, ER	40 mAU/mL, 21.0 ng/mL	a. Tumor marker for HCC irrelevant with AFP b. Combined with AFP to improve prediction efficiency
HBV DNA	*A, B*	OS, RFS, Liver failure	200IU/mL, 2000 IU/mL, 20,000 IU/mL, 10^4^ copies/mL, 30×10^6^copies/g (intrahepatic)	a. Level is not constant b. Affected by antiviral treatment c. Available in primary or repeat hepatectomy, curative or partial hepatectomy d. Postoperative HBV reactivation
HBsAg	*A, B*	OS, RFS, DFS, LR	200 IU/mL, 200 ng/mL, 1000 IU/mL, 4,000 IU/mL, IHC positive in tissues adjacent to cancer	a. Available in low HBV DNA level b. Some reports consider it irrelevant to RFS
HBeAg	*A*	OS, RFS	Positive serum HBeAg	a. HBeAg seroconversion b. No standardized HBeAg quantification yet
cfDNA	*A*	RFS, ER	18.71 ng/mL (Base on ER)	a. Influence by HBV-related HCC, HBV infection, liver function and other clinical parameters b. Diagnosis and monitoring of tumors
WFA^+^-M2BP	*A*	Recurrence	1.20, 1.80, 2.14	a. Liver fibrosis marker and predictor of HCC b. Vary COI in different studies
CA19-9	*A, B*	OS, RFS	39 U/mL, IHC positive	a. Common tumor marker of CCA in liver cancer b. Effective prediction in AFP (+) or (-) c. Secreted by the background liver, but not by tumor cells
POSTN	*A*	OS, RFS	36.8 ng/mL	a. Related to tumor progression, invasion and metastasis b. Better differentiating between HCC and non-malignant liver diseases combined with AFP
Interleukin	*A*	OS, RFS	IL-25 14.9mg/L, IL-6 3.1 pg/mL	a. Associated with occurrence, progression, immunosuppression and metastasis b. Measured *via* ELISA or ECLIA
CRP	*A, B*	LR, ER	1.5mg/dL	a. Non-specific inflammatory marker b. Related to the carcinogenesis, invasion and metastasis
GGT	*A, E*	OS, RFS	81.5 IU/L	a. Index of liver dysfunction b. Related to tumorigenesis
LDH	*A*	OS, RFS	203.5 IU/L	a. Metabolic enzyme b. Related to tumor initiation and progression
ALP	*A*	OS	136.5 IU/L	a. Hydrolase enzyme b. Diagnosis of hepatobiliary and bone diseases
GPR	*A, E*	OS, DFS	0.35	a. GPR = Gamma-glutamyl transpeptidase/Platelet (10^9)
NLR	*A*	OS, RFS	2	b. NLR = absolute neutrophil count/absolute lymphocyte count c. Effective prediction, regardless of AFP level
Monocyte count	*A*	OS, DFS	545/mm^3^	a. Preoperative peripheral blood b. Especially related to recurrence of HBV-related HCC
NANOG	*A, B*	OS, DFS, Recurrence	IHC Positive, Nanog 6.7 (Peripheral Blood)	a. Transcription regulator b. CSCs marker
AKT	*B*	OS	Phosphorylated, IHC positive	a. Key kinase in PI3K signal pathway b. Related to tumor initiation and progression
MACC1	*B*	OS, TFS	MACC1 mRNA (0.006732), IHC positive	a. Related to metastasis and invasion in solid cancers b. Expression or co-expression with c-MET as predictor
CK19	*B*	OS, DFS, RFS, ER	IHC positive	a. The least acidic type I cytokeratin, 40kDa b. Tumor prognostic and metastatic marker
Cirrhosis	*B*	OS, RFS	Pathologic features	a. Benefits from antiviral therapy b. Necessity of subgroup analysis in HCCs with and without cirrhosis
MVI	*B*	OS, RFS, Recurrence, Metastasis	Pathologic features	a. Available in postoperative specimen b. One of the characteristics of poor prognosis
LncRNA	*B, E*	RFS, OS	MSC-AS1, POLR2J4, EIF3J-AS1, SERHL, RMST, PVT1	a. Combination into prediction models b. Various types and functions
ALBI	*A, E*	Hepatic function	-2.28	a. formula = [log_10_ bilirubin (μmol/L) × 0.66] + [albumin (g/L) × (–0.085)] b. grades 1, 2, 3 = ≤ –2.60, > –2.60 to ≤ –1.39, > –1.39,
FibroScan	*C*	Ascites, Liver failure,	15.6kpa (ascites), 14.3kpa (liver failure)	a. Rapid, noninvasive, and reproducible method
LSM	*C, E*	CCI, ER, LR, OS, RFS	3.07 kPa (CCI threshold of 26.2), 5.5 kPa (ER), 3.62kPa (LR), 13.2 kPa	a. Better prediction of postoperative complications by MRE than TE b. Reported as a risk factor of poor prognosis with different value
ST	*C*	Liver failure, morbidity, mortality	43.5 mm	a. Noninvasive indicators detected by varied measuring instruments
TS	*C*	ER	1kda increase related 16% recurrence	a. Noninvasive, quantitative predictor
18F-FDG PET/CT	*C*	OS	Positive	a. Unrelated to RFS
T2DM	*D*	OS, RFS	Diagnosis	a. Independent risk factor of MVI
Age	*D*	Complications, Mortality	Age of 65, 70	a. Not contraindication for hepatectomy b. Well-controlled preoperative comorbidities are recommended
ICG-R15	*D*	Complications, Liver failure	Grouped by 50% and 85% percentile of ICG-R15(≤ 4.6%, 4.6–9.9%, > 9.9%)	a. Linear predictor
CCI	*E*	Complications	26.2	a. Better predictive performance combined with other indexes b. Available at www.assessurgery.com
γ-GT/ALT	*A, E*	OS, DFS	1.52	a. Hepatic inflammatory index
APRI	*A, E*	OS, DFS	0.62	a. APRI = [(AST/ULN) × 100/PLT (10^9^/L)]
CONUT	*A, E*	OS, RFS, HBV reactivation	2, 3	a. Scoring system in **Table 4** b. Available in low HBV-DNA level

a Predictors of worse prognoses: Expression, existence, or higher level of the factor is related to worse prognoses, including worse OS, RFS, DFS, recurrence, more complications, and others.

b Classification: A: Serological and hematological predictors, B: Genetic, pathological predictors, C: Imaging predictors, D: Other predictors, E: Analysis models and indexes as predictors.

c Specific methods details in references.

**Table 2:** Predictors of better prognoses.

Predictor	Classification	Related prognosis	Reference COI/threshold/value	Notes
Antiviral therapy	*D*	HBV reactivation, MVI, OS, RFS	Used pre- and/or post-operatively	a. Related to HBV DNA level closely b. Beneficial to even low preoperative HBV DNA
Prealbumin	*A*	Short-term mortality, morbidity	17 g/dL	a. Reflecting liver function and nutritional status b. Preoperative evaluation
LMR	*A*	OS, RFS	3.23	a. LMR = absolute lymphocyte count/absolute monocyte count b. No statistical significance in non-cirrhotic HCC
HPSE	*B*	OS, ER	DNA copy number 2, IHC positive	a. Enhanced tumour growth and metastasis
sFLR	*C, E*	Liver failure, Morbidity, Mortality	53.8%	a. sFLR = FLR/eTLV, b. eTLV (cm^3^) = 706.2×BSA (m^2^) + 2.4 c. BSA (m^2^) = 0.00607 × height (cm) + 0.0127 × weight (kg) - 0.0698 (for men) d. BSA (m^2^) = 0.00586 × height (cm) + 0.0126 × weight (kg)-0.0461 (for women) b. Stronger predictor combined with ALBI (sFLR × ALBI)
IVIM	*C, E*	Recurrence	D-value (0.985 × 10^−3^ mm^2^/s)	a. Noninvasive, no contrast agent preoperative predictor
PNI	*A, E*	OS, DFS	48.5	a. Systemic inflammatory index b. PNI = 10 × serum albumin value (g/dL) + 0.005 × lymphocyte count in peripheral blood

a Predictors of better prognoses: Expression, existence, or higher level of the factor is related to better prognoses, including better OS, RFS, DFS, recurrence, fewer complications, and others.

b Classification: A: Serological and hematological predictors, B: Genetic, pathological predictors, C: Imaging predictors, D: Other predictors, E: Analysis models and indexes as predictors.

c Specific methods details in references.

**Table 3:** Predictors of variable prognoses.

Predictor	Classification	Related prognosis	Reference COI/threshold/value	Notes
Tregs, Bregs	*A*	Clinical features of HCC	Postoperative elevation	a. Tregs: Correlated with HBV infection history, inversely related to hepatic vein invasion, PVTT, ferritin, BCLC score, and others. b. Bregs: Correlated with HBeAg, HBV DNA c. Level lower than healthy individuals and patients with CHB
Mutations and Genetic variability of HBV genome	*A, B*	OS, RFS	Gene mutations, Proofreading deficiencies, Different genotypes	a. Four ORF: Pol, Pre-S/S, Pre-C/C, HBx b. Pol: 31(Ser314Pro), 529(Asp480Asn), 1078(Ser663Ala) c. PreC/C: 1915, 2134, 2221, 2245, 2288 sites d. HBx: 1383, 1461, 1485, 1544, 1613, 1653, 1719, 1753, 1527, 1637, 1674, 1762/1764 e. Three HBx genotypes both in tumor and nontumor tissue: HBx-EHBH1, HBx-EHBH2, HBx-EHBH3 f. HBV core promoter: A1762T/G1764A g. cccDNA: T1719G, C1329A, T3098C

a Predictors of variable prognoses: Expression, existence, or level of the factor is related to variable prognoses.

b Classification: A: Serological and hematological predictors, B: Genetic, pathological predictors, C: Imaging predictors, D: Other predictors, E: Analysis models and indexes as predictors.

c Specific methods details in references.

## 2 Serological and hematological predictors

The pathogen of HBV belongs to the Hepadnaviridae family, with DNA as genetic material. The genetic material or components of HBV can be found in the infected individuals’ blood and liver tissue. The pathogenic mechanisms of HBV infection have been thoroughly researched (6, 7). The natural history of HBV-related HCC generally includes HBV infection, failure to trigger innate immunity, slow and weak adaptive immunity resulting in virus persistence, chronic hepatitis, fibrosis, cirrhosis, and eventually deterioration causing HCC (8).

### 2.1 AFP, DCP

Alpha-fetoprotein (AFP) is considered an important biomarker for early screening and diagnosis of HCC, so its prognosis prediction for hepatectomy is also of concern. Because of the dynamic changes in serum levels, AFP can serve as a stratification factor and prognostic predictor for treatment selection and recurrence prediction. A prospective study included 189 HBV-related HCC patients between 1995-2008 found that patients with preoperative AFP in the first quintile (1.4-4.1 ng/mL) had better overall survival (OS) and disease-free survival (DFS) than the other four groups (9). A retrospective study regarding 400 ng/mL as a cut-off value for AFP, demonstrated that AFP concentration higher than 400 ng/mL was an independent risk factor for shorter DFS and OS after HBV-related HCC hepatectomy, while its sensitivity and specificity were low (10).

In the surveillance and early diagnosis stage of HCC, des-γ-carboxy prothrombin (DCP) usually acts as a supplementary predictor to AFP (11). Given the low prognostic predictability of AFP, DCP can also be used as a complementary predictor of HBV-related HCC undergoing curative resection combined with AFP preoperatively. A retrospective analysis suggested that the AFP cut-off value for early recurrence (ER, within 2 years) after primary hepatectomy was 529.8 ng/mL, and DCP was 21.0 ng/mL (12). Another retrospective analysis suggested that high levels of AFP (≥20 ng/dL) and DCP (≥40 mAU/mL) were independent risk factors for tumor relapse and DFS in patients with HBV-related HCC after curative resection (13).

### 2.2 Serum HBV DNA and antiviral therapy

The detection of HBV DNA in serum is a significant biomarker of HBV infection and the most frequently used prognostic predictor of HBV-related HCC. Preoperative HBV DNA level [≥20,000 IU/mL (14), >10^4^ copies/mL (15)] is associated with ER in patients with HBV-related HCC undergoing radical hepatectomy. The level of HBV DNA in patient’s serum often changes with the progression of the disease. Thus, the level of HBV DNA is not constant, which means that dynamic detection of HBV DNA level is meaningful. Analyzing HBV DNA levels before primary and repeated hepatectomies, a study found that when HCC relapses, low levels of HBV DNA suggest better prognoses, including primary and repeat hepatectomies (16). Preoperative low-level viremia (HBV DNA <2000 IU/mL) was associated with longer HCC recurrence intervals, and HBV DNA level ≥100 IU/mL was one of the independent risk factors for HBV-related HCC relapse (17). After hepatectomy for HBV-related HCC, HBV infection may persist in liver tissue, and liver dysfunction and even recurrence of liver cancer may occur. Preoperative HBV DNA level is related to the incidence of postoperative complications, especially the incidence of postoperative liver failure (18). Some research demonstrated that HBV-related HCC in patients with low preoperative HBV load (<2000 IU/mL) could emerge HBV reactivation after curative or partial hepatectomy (19, 20). In patients with HCC who underwent curative hepatectomy for non-cirrhotic liver, the prognosis of patients with HBV infection was worse than patients without HBV infection (21).

From the above studies, it can be inferred that preoperative use of antiviral therapy to reduce the preoperative HBV DNA level may be beneficial to improve the prognosis of patients with HBV-related HCC. A meta-analysis concluded that low viral load HBV-related HCC receiving antiviral therapy after curative hepatectomy could reduce viral reactivation and improve OS (22). HBV reactivation, which is associated with HCC recurrence, will occur in patients with HBV-related HCC after hepatectomy, even in those with undetectable HBV DNA before surgery. Therefore, some studies suggested that even if HBV DNA was not detected before surgery or HBV was reactivated after surgery, antiviral therapy should be used to reduce HBV reactivation and improve the prognosis after re-hepatectomy (23–25). In a multicenter retrospective study of patients with HBV-related HCC who underwent radical hepatectomy in China, it was suggested that postoperative antiviral therapy was an independent protective factor for 10-year survival rate (26). In addition to the direct effect of HBV on HCC progression, high serum HBV levels can also lead to poor outcomes in patients with HBV-related HCC by the association with microvascular invasion (MVI) (27). Antiviral therapy can be divided into preoperative and postoperative treatment. A retrospective cohort study suggested that a high preoperative HBV load is an independent risk factor for MVI, and that over 90 days of antiviral treatment before partial hepatectomy was associated with MVI reduction and lower early tumor recurrence rate (28). A randomized controlled trial demonstrated that after R0 hepatectomy for patients with preoperatively low serum HBV DNA (<2000 IU/mL), started receiving antiviral therapy (telbivudine tablets) 4-7 days after surgery could markedly reduce HCC recurrence (29).

### 2.3 HBsAg, HbeAg, cfDNA

Corresponding to HBV DNA, one of the multiple risk factors for late recurrence (LR, over 2 years) in patients with HBV-related HCC is preoperative HBsAg levels (≥4,000 IU/mL) (14). A retrospective analysis of 881 patients with HBV-related HCC who underwent curative hepatectomy, showed that preoperative and postoperative low level of HBsAg (<200 ng/mL) was correlated with better prognoses, while antiviral treatment might be beneficial to a high level of HBsAg (≥200 ng/mL) (30). Retrospective research in Taiwan suggested that HBsAg >200 IU/mL was an independent predictor of late recurrence (years 2 to 5), and HBsAg >50 IU/mL was an independent predictor of late mortality (over 5 years) (31). Meanwhile, HBsAg could serve as a monitor of antiviral therapeutic effect when HBV DNA level was low (<2000 IU/mL) (17). Original Investigation by Gang Huang, et al. demonstrated that preoperative serum HBsAg level over 1000 IU/mL was one of the independent risk factors for HCC recurrence in patients with low HBV DNA load (<200 IU/mL) receiving partial hepatectomy (32).

HBeAg seroconversion is common, and its quantification is not yet standardized (6). HBeAg level can also be down-regulated by peginterferon treatment and can be served as an efficacy indicator of the latter (33). Thus, the rate of HBeAg-positive patients with HCC is not high after a long progression from HBV infection. Nevertheless, a propensity score matching analysis about HCC undergoing hepatectomy and serum HBeAg suggested that positive serum HBeAg had a negative impact on tumor relapse and long-term survival (34).

Other detectable indicators in the blood may serve as preoperative and postoperative predictors, apart from the HBV particle component. Circulating cell-free DNA (cfDNA), which is highly fragmented DNA, exists in human blood circulation and is free from cells. Many studies have shown that the concentration of cfDNA was higher in the blood of cancer patients compared with healthy individuals (35), and a later study suggested that total plasma cfDNA levels could be used as a biomarker to predict ER of HBV-related HCC (36).

### 2.4 Protein in blood

#### 2.4.1 WFA^+^-M2BP

Studies have reported that Wisteria floribunda agglutinin-positive Mac-2-binding protein (WFA**^+^-**M2BP) was highly correlated with the development of hepatocellular carcinoma after HBV infection, whether or not receiving nucleos(t)ide analogues treatment (37, 38). A study from Korea showed that postoperative serum WFA**^+^-**M2BP was an independent risk factor of HBV-related HCC recurrence after curative hepatectomy, with cut-off value 2.14 obtaining maximized sensitivity and specificity (area under receiver operating characteristic curve, AUC = 0.632, *P* = 0.010) (39).

#### 2.4.2 CA19-9

Carbohydrate antigen 19-9 (CA19-9) is generally considered a tumor marker of cholangiocarcinoma (CCA) (40). A study targeting HCC suggested that CA19-9 ≥39 U/mL predicted worse OS and RFS regardless of whether AFP was positive or negative in HBV-related HCC patients who underwent curative resection (41). Elevated CA19-9 levels in patients with HCC are a rare and interesting occurrence, as it is more common in cholangiocarcinoma, which is also a type of primary liver cancer. A recent study found that combined hepatocellular-cholangiocarcinoma (cHCC-CCA) is a rare primary liver cancer with clinical and pathological features of HCC and CCA (42). Half of cHCC-CCAs a may be misclassified as HCC. When CA19-9 levels are elevated, it is necessary to distinguish between HCC, CCA, or cHCC-CCA. Therefore, the predictive role and diagnostic value of CA19-9 cannot be ignored in clinical practice.

#### 2.4.3 POSTN

Periostin (POSTN) is an extracellular matrix protein. In addition to being related to allergic diseases and inflammatory diseases, more and more studies have shown that POSTN is abnormally expressed in a variety of cancers and related to the invasion and metastasis of tumor cells (43–45). Preoperative serum POSTN measured by enzyme-linked immunosorbent assay (ELISA) showed that high-level serum POSTN was associated with HCC progression and can serve as an independent worse prognostic predictor for OS and RFS (46). Detecting POSTN expression levels through immunohistochemistry (IHC) staining can reveal similar results, and combined with microvascular invasion can serve as an independent predictor for prognosis (47).

#### 2.4.4 Interleukins

Members of the interleukin family are a class of cytokines produced by and act on a variety of cells in many ways. Studies indicated that abnormal expression of interleukin family members was associated with HBV-related HCC in initiation, progression, immunosuppression, and metastasis (48–50). A study that enrolled consecutive patients with HBV-related HCC who underwent hepatectomy from 2008 to 2015 suggested that IL-25 level ≥14.9 mg/L was an independent preoperative predictor of worse OS and RFS after liver surgery (51). Preoperative serum IL-6 level above 3.1 pg/mL was related to lower OS and RFS rates. When the level exceeded 3.7 pg/mL, a saturation effect occurred (52).

#### 2.4.5 CRP

There are some other routine serological indicators tested before hepatectomy. C-reactive protein (CRP) is a non-specific inflammatory marker. Studies have shown that CRP overexpression verified by ELISA and IHC was related to the carcinogenesis, invasion, and metastasis of HBV-related liver cancer (53, 54). A retrospective study suggested that, in patients with HBV-related HCC, the saturation effect for preoperative serum CRP existed in 2.1 mg/dL, and the best cut-off value for CRP predicting ER and LR was 1.5 mg/dL (55).

#### 2.4.6 Prealbumin

Prealbumin, a small, short half-life protein synthesized by hepatocytes, is more sensitive than albumin when hepatocytes are damaged. An analysis from a Chinese multicenter database showed that patients in a group with low preoperative serum prealbumin level (17 mg/dL as cut-off value) had higher postoperative morbidity, mortality, and hepatic insufficiency (56).

#### 2.4.7 GGT, LDH, ALP

A retrospective analysis for HBV-related HCC underwent curative liver resection found that gamma-glutamyl transpeptidase (GGT) and lactate dehydrogenase (LDH) were important independent prognostic factors of OS and RFS, while alkaline phosphatase (ALP) was an important independent predictor of OS, not RFS, with cut-off value 81.5 IU/L, 203.5 IU/L, 136.5 IU/L, respectively (57).

### 2.5 Ratios in blood

#### 2.5.1 GPR

The gamma-glutamyl transpeptidase to platelet ratio (GPR) has been reported as an accurate non-invasive predictor for HCC development in patients with HBV infection (58). Combined with the preoperative serum fibrinogen index, GPR could better predict the prognosis of HBV-related HCC underwent hepatectomy as an effective non-invasive predictor (59).

#### 2.5.2 NLR

As one of the most important organs in human blood circulation, the physiological function of the liver can be reflected in routine blood examinations. A meta-analysis including 17 studies demonstrated that preoperative Neutrophil-Lymphocyte ratio (NLR, the ratio of absolute neutrophil count to absolute lymphocyte count) was highly correlated with the prognosis of HCC (60). A report showed that preoperative peripheral NLR >2 was an independent risk factor of worse OS and DFS for patients with HBV-related HCC, regardless APF is normal or not (61).

#### 2.5.3 Monocyte, LMR

As a parameter for evaluating inflammation in routine blood tests, high-level preoperative peripheral blood monocyte (≥545/mm^3^) has been reported as an independent risk factor for HCC undergoing hepatectomy, especially with HBV infection (62). Combined with absolute peripheral blood lymphocyte and monocyte counts, a low preoperative lymphocyte-to-monocyte ratio (LMR, <3.23) has been proven to serve as an independent prognostic factor in patients with HBV-related HCC who received curative resection (63).

### 2.6 Tregs, Bregs

A case-controlled study enrolled 36 patients with HCC, six patients with CHB as a control group, demonstrated that frequencies of peripheral regulatory T cells (Tregs) and regulatory B cells (Bregs) uplifted after liver resection. The research suggested that anti-Tregs and anti-Bregs combination treatments could improve the postoperative prognosis of HBV-related HCC (64).

## 3 Genetic, pathological predictors

Various test samples and indicators of HBV-related HCC patients are available clinically. The genetic and pathological factors can serve as predictors besides the indicators in serum (65).

### 3.1 Mutations and genetic variability

The HBV genome encodes the following four proteins with overlapping open reading frames (ORF): the viral polymerase (Pol), the envelope protein (Pre-S/S), the core protein (Pre-C/C), and a multifunctional nonstructural protein called X (HBx) (7, 66, 67). Previous studies have confirmed that proofreading deficiencies of the HBV genome affect the occurrence, development, and prognosis of HBV-related HCC. The mutations at sites 31(Ser314Pro), 529(Asp480Asn), and 1078(Ser663Ala) in Pol were identified as independent predictors of survival in patients with HBV-related HCC after hepatectomy (68). The mutations of the PreC/C region at 1915, 2134, 2221, 2245, and 2288 sites have been reported as significant independent predictors of worse survival in patients with HCC who underwent tumor resection. Similar mutations related to postoperative survival in HCC were also present in the X gene (69, 70). A variety of HBX gene sequences that encode a 17kDa protein have been observed. A study by Qing-guo Xu, Hui Liu et al. demonstrated that three new genotypes, HBx-EHBH1 (HBx-E1), HBx-EHBH2 (HBx-E2), and HBx-EHBH3 (HBx-E3) could be identified in both the tumor and non-tumor tissues for patients with HBV-related HCC after liver resection. Among these genotypes, HBx-E2 detected from tissue or serum predicted better prognoses of patients with the intermediate stage (Barcelona Clinic Liver Cancer, BCLC stage B) HCC (71).

### 3.2 HBV DNA, rcDNA, LncRNA, HBsAg

Another study analyzed HBV DNA in noncancerous part of HBV-related HCC tissue after tumorectomy and found that HBV basal core promoter mutation, like A1762T/G1764A mutation, as well as the amount of HBV DNA, were independent predictors for both OS and RFS (72).

The genetic material relaxed circular DNA (rcDNA) in the HBV nucleocapsid which exists in the cytoplasm is repaired to cccDNA after being transported into the nucleus as an episomal transcriptional template (7). The full-length cccDNA sequences of tumor and non-tumor tissues in HBV-related HCC patients from liver biopsy samples found that mutations T1719G, C1329A, and T3098C were related to different survival after surgery (73).

Long non-coding RNA (lncRNA) is a non-coding RNA with a length of more than 200 nucleotides, which is a research hotspot in epigenetics and oncology over the past decade. Studies have demonstrated the potential of lncRNAs as biomarkers for predicting and diagnosing HBV-related HCC and therapeutic targets (74, 75). A study based on Gene Expression Omnibus and The Cancer Genome Atlas databases constructed a prognostic nomogram combining the lncRNA signature and clinical characteristics showed that risk model with MSC-AS1, POLR2J4, EIF3J-AS1, SERHL, RMST, and PVT1 upregulated in HBV-related HCC tissues was significantly correlated with poor RFS (76).

In patients with HCC after a long period of HBV infection, the HBsAg level in serum does not remain unchanged because of seroclearance. There is a question as to whether HBsAg exists in tumor tissue or liver tissue, which can serve as a stable index. A study testing HBsAg expression by IHC in liver tissue microarrays which collected tissue approximately 2 cm away from the tumor after hepatectomy suggested that the HBsAg-positive group had a better OS than the negative group, but RFS was not statistically significant. HBsAg expression in liver tissue was an independent risk factor for patients with HBV-related HCC who underwent surgery (77).

### 3.3 Protein mutations and expression in tissues

#### 3.3.1 AKT

AKT, a key kinase in PI3K signal pathway, is an important protein in cancer initiation and progression, so it is often used as an indicator in cancer research (78, 79). A study on double mutation A1762T/G1764A (TA) combined with other mutation(s) (TACO) and AKT showed that overexpression of TACO in HBV genome core promoter and phosphorylated AKT (pAKT) in HBV-related HCC tissues were independent predictors of poor prognosis after hepatectomy (80).

#### 3.3.2 NANOG

NANOG is a critical transcription regulator of embryonic stem (ES) cells with self-renewal and multilineage differentiation (81). Cancer stem cells (CSCs) share similar features with ES cells. Thus, NANOG has been reported to play an important role in CSCs’ abilities of stemness, unlimited self-renewal, metastasis, invasiveness, angiogenesis, and drug resistance (82). Numbers of NANOG-positive cells measured in peripheral blood (Nanog >6.7) and HCC tissues (positive-IHC) from patients, 90% of whom with HBV infection, showed a positive correlation to the worse prognosis for HCC patients after R0 resection (83).

#### 3.3.3 HPSE

High expression of heparinase (HPSE) has been reported in a variety of cancers and is also regarded as a significant regulator in the tumor microenvironment (84). The loss of heterozygosity, mRNA level, and protein expression of HPSE detected by SNP microarray, quantificational reverse transcription-polymerase chain reaction, and IHC, respectively, demonstrated that genetic alteration and decrease of HPSE expression were related to the poor outcome of HBV-related HCC (85).

#### 3.3.4 MACC1

As the term suggests, metastasis-associated in colon cancer 1 (MACC1) is generally correlated with the metastasis of colon cancer. Nevertheless, MACC1 has been reported to be associated with metastasis and invasion in multiple solid cancers after a decade of research (86–88). A retrospective study enrolled 354 patients with HBV-related HCC who underwent surgical resection and suggested that a high expression level of MACC1 in the tumor was correlated with poor prognosis and progression of HCC (89).

#### 3.3.5 CK19

Cytokeratin 19 (CK19), with a molecular weight of 40kDa, is the least acidic type I cytokeratins (CKs) which has been considered a tumor prognostic and metastatic marker in varied cancers (90). A meta-analysis including 17 studies with 2943 patients showed that CK19 overexpression which was detected by IHC or IHC plus tissue microarrays was associated with HCC early recurrence and lower survival rate (91). A retrospective study based on two institutions’ data plotted nomogram of 2-year RFS and analyzed survival risk factors by Cox regression model showed that CK19 expression in tumor tissue detected by IHC after hepatectomy was an independent adverse factor for HBV-related HCC recurrence (92).

### 3.4 Cirrhosis

Liver cirrhosis is not inevitable in the development of HBV-related HCC. A study using the modified Ishak method to evaluate liver fibrosis of HBV-related HCC specimens after hepatectomy demonstrated that cirrhosis (Ishak stage 6) was an independent predictor of worse OS and RFS (93, 94). A long-term single-center follow-up study conducted in Japan from 1995 to 2013 showed differences in prognostic factors between HCC with cirrhosis (HCC-C) and HCC without cirrhosis (HCC-NC), and it was recommended an emphasis subgroup analysis of the two to achieve individualized treatment. The analysis suggested that the long-term prognosis of HCC-NC patients was better than that of HCC-C patients, and anti-viral therapy might be beneficial to HCC, especially HCC-C patients (95).

### 3.5 MVI

Microvascular invasion (MVI) generally is regarded as a predictor that reflects the tendency of invasion and metastasis causing recurrence or distant metastasis of HCC (96). A multicenter study from China that included 1517 HCC patients with MVI showed that the actual 5-year survival rate is 33% after hepatectomy, and some patients received other treatments after surgery (97). Another retrospective study from China included 2,508 patients with a solitary HBV-related HCC, suggesting that a wide resection margin is conducive to a better long-term prognosis for 929 patients with MVI (98). Because MVI can only be clearly diagnosed in postoperative specimens at present, follow-up treatment is necessary to improve long-term survival. Research about recurrent HBV-related HCC demonstrated that MVI (–) patients receiving re-resection or RFA could obtain longer survival than TACE, while no difference in MVI (+) patients (99).

## 4 Imaging predictors

With the development of imaging techniques and instruments, such non-invasive methods have been playing important roles in predicting the prognosis of patients preoperatively and postoperatively. Primary liver cancer is a type of cancer that can be clearly diagnosed through imaging and clinical manifestations in order to enter the treatment process as soon as possible. Therefore, imaging-related factors are important predictors that cannot be overlooked.

### 4.1 CT

Computed Tomography, abbreviated as CT, is a commonly utilized imaging method for HCC diagnosis. A retrospective study enrolled 229 patients with three-dimensional CT reconstruction before curative hepatectomy demonstrated that albumin-bilirubin (ALBI) grade, which had been reported as a new assessment tool for hepatic function (100, 101), combined with standardized future liver remnant (sFLR) measured by CT could better predict prognosis of HBV-related HCC than separate indicators (102).

### 4.2 US

Liver ultrasound (US) combined with AFP are common methods for early screening and diagnosis of HCC. As a non-invasive ultrasonic examination, transient elastography (TE) is often used to measure liver fibrosis, cirrhosis, and stiffness, which are important reference factors for determining whether surgery can be performed and evaluating the possibility of liver failure after surgery. A prospective study applied TE assessed by FibroScan demonstrated that FibroScan value one week before surgery was an independent risk factor for predicting postoperative ascites, and liver failure, with a best cut-off value of 15.6 kpa, and 14.3 kpa, respectively (103). Besides liver stiffness measurement (LSM), a retrospective analysis found that splenic thickness (ST) measured by US was significantly associated with post-hepatectomy liver failure and an independent risk factor of postoperative morbidity and mortality in patients with HBV-related HCC (104).

### 4.3 MRI

It is reported that another implication for LSM by magnetic resonance elastography (MRE) based on magnetic resonance imaging (MRI) has better performance in detecting fibrosis and better postoperative prediction (evaluated by comprehensive complication index, CCI) in HCC than TE (105). A prospective study that collected HCC patients who were under Milan criteria and received MRE before treatment, including hepatectomy, RFA, or TACE showed that LSM by MRE could serve as an independent predictor of ER in patients with HCC under Milan criteria after treatment (106). LSM by MRE as a parameter of preoperative and postoperative prediction models for ER indicated a good predictive performance and served as an independent predictor of LR in HBV-related hepatocellular carcinoma patients (107). A study enrolling 263 patients with HBV-related HCC who underwent curative resection suggested that preoperative LSM ≥13.2 kPa was an independent risk factor causing worse OS and RFS (108). Another prospective study measured HCC tumor stiffness (TS) measured by MRE in patients with HBV infection before hepatectomy (median:6 days, range:1-30 days) suggested that TS and vascular invasion were risk factors of HCC ER analyzed by the multivariate Cox proportional hazard model (109). Intravoxel incoherent motion (IVIM) is a derivative model of diffusion-weighted imaging, which can assess water molecule diffusion and blood perfusion, and display both perfusion and diffusion information at the same time. Because no contrast medium is required, IVIM-based MRI has become a research hotspot in the imaging field related to tumors in recent years (110). D-value (true diffusion coefficient, <0.985 × 10^−3^ mm^2^/s) of tumors in HBV-related HCC patients obtained from preoperative IVIM models was reported as a risk factor for tumor recurrence after hepatectomy (111).

### 4.4 PET/CT

Positron emission tomography/computed tomography (PET/CT) is a combination of PET and CT, using F-18-fluoro-2-deoxy-glucose (18F-FDG) to reflect cellular metabolism, which can be used to diagnose tumors, neoplasm staging, and discover metastases. Studies have suggested that 18F-FDG PET/CT could be beneficial to the diagnosis of HCC metastasis and recurrence (112). Appropriate consistency of PET and CT texture features as 18F-FDG PET/CT radiomics signature could serve as an independent marker for estimation of MVI and DFS in patients with very-early- and early-stage HCC (113). A study involving patients with HBV-related HCC who received 18F-FDG PET/CT detection preoperatively between 2007 to 2014 showed that positive result is not related to recurrence but better OS (114).

## 5 Other predictors

In addition to various examination indexes, the patient’s general condition and basic characteristics before surgery are also effective predictors for preoperative evaluation of operation risk and prognosis, preoperative antiviral treatment mentioned above for instance.

### 5.1 T2DM

It is generally acknowledged that MVI is a risk factor for metastatic tumor recurrence in HBV-related HCC leading to poor prognosis (96). A multicenter retrospective study in China found that HBV-associated HCC patients with type 2 diabetes mellitus (T2DM) had a higher risk of MVI occurrence, while non-T2DM patients had better OS and RFS (115). Since MVI can be only defined in postoperative specimens at present, incorporating preoperative T2DM evaluation is beneficial for establishing earlier predictive models and improving surgical protocols.

### 5.2 Age

The age of HCC patients is also one of the important characteristics to predict the prognosis. A study enrolled HCC patients who underwent hepatectomy at Sun Yat-sen University Cancer Center from 1983 to 2006 suggested that HCC in the elderly (≥70 years) was less associated with HBV, less advanced, and less aggressive, compared with younger patients. Furthermore, the study concluded that pathological tumor-node-metastasis staging was the only independent predictor of OS in the elderly (116). Another retrospective study with a cut-off age of 65 years showed that compared with younger patients (<65 years), the elderly patients with HBV-related HCC had lower positive rates of serum HBsAg and HBeAg, lower levels of HBV DNA, but similar morbidity and levels of complications (117). Therefore, the above research indicated that age should not be considered a contraindication of hepatectomy. Hepatectomy is a safe and effective treatment for elderly patients without or with controllable complications before surgery.

### 5.3 ICG-R15

The indocyanine green retention rate at 15 min (ICG-R15) is widely used in the clinic to evaluate liver function reserve before hepatectomy (118). A retrospective study that enrolled 354 HBV-related HCC patients who underwent initial hepatectomy suggested that preoperative ICG-R15 could better assess postoperative major complications and severe post-hepatectomy liver failure than Child-Pugh, MELD, and ALBI scores (119).

## 6 Analysis models and indexes

In addition to a single factor, some studies have found that analysis models or indexes combined with multiple single factors can better predict the prognosis of HBV-related HCC. This review adopts the relevant evaluation models, scoring models, and indexes that have been widely approved and applied.

### 6.1 CCI

Comprehensive Complication Index (CCI), which is based on the Clavien-Dindo classification (120) (www.assessurgery.com) can serve as an evaluation index combining all postoperative complications (121). A multicenter retrospective study suggested that models based on preoperative and postoperative CCI combined with other parameters could establish a more accurate prediction statistical model for patients with HBV-related HCC (122).

### 6.2 Inflammatory biomarkers

Systemic and hepatic inflammatory biomarkers have been reported as potential prognostic predictors for HBV-related HCCs. A study of systemic inflammatory markers and hepatic inflammatory markers, including neutrophil-to-lymphocyte ratio (NLR), platelet-to-lymphocyte ratio (PLR), Prognostic Nutritional Index (PNI) (123), and aspartate aminotransferase-to-platelet ratio index (APRI), γ-glutamyl transferase (γ-GT)/alanine aminotransferase (ALT), respectively, showed that PNI and γ-GT/ALT were independent predictors of prognosis in patients with HBV-related HCC (124). (Formulations shown in **Table 1**, **2**) The inflammation biomarkers mentioned above contain multiple indexes including platelet. Abnormal changes in peripheral platelet are usually correlated with liver diseases. A retrospective study of platelet-based indexes, including platelet count, PLR, and especially APRI indicated that the elevation of platelet-related inflammatory indicators was associated with cirrhosis and HBV infection, predicting worse OS and DFS (125).

### 6.3 COUNT

The controlling nutritional status (COUNT) score is a scoring model for nutritional status proposed in 2005 by combining serum albumin, total cholesterol concentration, and total lymphocyte count (126). COUNT score is used to predict the prognosis of various tumors. Due to the included parameters related to liver nutritional function and HBV infection, COUNT can effectively serve as a prognostic factor for HBV-related HCC. It was reported that the preoperative COUNT score was a significant independent predictor of OS and an effective indicator of postoperative HBV reactivation in patients with HBV-related HCC (127, 128) (scoring system shown in **Table 4**).

**Table 4:** COUNT scoring system.

Variables	Nutrition status (score)			
Serum albumin (g/dL)	≥ 3.50 (0)	3.00-3.49 (2)	2.50-2.99 (4)	< 2.50 (6)
Serum total cholesterol (mg/dL)	≥ 180 (0)	140-179 (1)	100-139 (2)	< 100 (3)
Total lymphocyte count (/ml)	≥ 1600 (0)	1200-1599 (1)	800-1199 (2)	< 800 (3)
CONUT score	Normal (0-1)	Mild (2-4)	Moderate (5-8)	Severe (9-12)

## 7 Extended content

The main risk factors for HCC include chronic viral infections (HBV, HCV, HIV), metabolic risk factors (metabolic syndrome, obesity, type II diabetes, and non-alcoholic fatty liver disease), consumption of aflatoxin-contaminated foods, and smoking (1, 129, 130). In addition to HBV-related HCC, HCV-related HCC, and metabolism-related HCC also account for a large proportion and have significant global and regional distribution differences. Therefore, in the prevention and treatment strategies of HCC, it is essential to comprehend the prognostic predictors of HCC related to other risk factors.

### 7.1 HCV-related HCC

Hepatitis C virus (HCV) is a major type of hepatitis, along with HBV, have been targeted in global health sector strategies for the period 2022-2030 (2). According to WHO statistics, an estimated 58 million people worldwide are infected with HCV. In 2019, about 290 000 people died from HCV, mainly due to cirrhosis and HCV-related HCC.

Numerous studies have shown that high HCV viral titer is associated with poor prognoses of HCV-related HCC (131, 132), which is similar to the relationship between HBV and HCC. HCV genotypes and serological clearance or not were reported to be unrelated to the long-term outcome of HCC (132, 133). WHO estimated about 15%-45% of individuals infected with HCV could occur spontaneous clearance without treatment. Unlike HBV, HCV is an RNA virus, which has a distinguished virus amplification mechanism. Therefore, the recommended antiviral therapy for HCV is direct-acting antiviral agents (DAAs). DAAs can result in nearly 100% sustained virological response (SVR), compared with approximately 50% with interferon-based treatments (134). Whether SVR is achieved preoperatively or postoperatively, it is associated with better prognoses of HCV-related HCC (135, 136). Further research suggested that preoperative SVR is more beneficial than postoperative SVR, with similar surgical outcomes but better postoperative liver function (137–139).

HCV-related HCC and HCV-related HCC have common prognosis predictors, such as AFP, DCP, fibrosis, cirrhosis, and diabetes mellitus (12, 140, 141). However, due to differences in tumor biology and characteristics baseline, the specific prediction results of predictors are not the same leading to different prognoses (142, 143). Therefore, in the study and treatment of HCC, patients with hepatitis virus infection need to be clearly classified and subgroup analyzed.

### 7.2 NBNC-HCC

Non-HBV non-HCV HCC (NBNC-HCC) refers to patients with HCC who have not detected biomarkers of HBV and HCV, respectively, negative HBsAg and hepatitis C antibody (HCVAb) (144). NBNC-HCC commonly progress from non−alcoholic fatty liver disease (NAFLD), non−alcoholic steatohepatitis (NASH, a severe form of NAFLD), alcoholic liver disease (caused by heavy alcohol intake), exposure risk factors (such as aflatoxin, smoking) and other etiologies (such as primary biliary cirrhosis, autoimmune hepatitis, Wilson disease, Budd-Chiari syndrome) (1, 145).

The common cause of NBNC-HCC in high-income countries is NAFLD, of which the primary risk factor is metabolic syndrome including central obesity, dyslipidemia, hypertension, and diabetes mellitus (146, 147). During surgical treatment, a stable metabolic state is a crucial factor in the perioperative period. A retrospective study about major liver resection suggested that metabolic disorders would increase the risk of perioperative death in HCC patients (148). Meanwhile, HCC patients with metabolic syndrome have specific risks of postoperative complications and long-term survival benefits after surgery which means the necessity of perioperative regulation (149, 150). COUNT scoring system can also be an independent predictor of NBNC-HCC with ≥4 meaning worse OS and RFS (151). Compared with HBV-related and HCV-related HCC, although NBNC-HCC has different tumor characteristics and may have advanced tumors, DCP can serve as a diagnostic and prognostic predictor on both sides (152, 153).

A community-based prospective longitudinal study in Taiwan which is an HBV-endemic and HCV-endemic area found that high-level triglyceride (>150 mg/dL) was a decreased risk factor for HCC (154). A Subgroup Analysis of HCC patients grouped with HBV-related HCC (HBsAg+), HBV-related HCC (HCVAb+), NBNC-related HCC (HBcAb+), and pure NBNC-related HCC demonstrated that NBNC-related HCC patients with a history of HBV infection might have better survival than other groups (155). A multicenter retrospective study in France demonstrated that in NBNC-HCC patients with non-cirrhotic liver, stratified prediction can be performed based on the number (≤2 or >2) and size (≤10 or >10 cm, if less than 2 nodules) of nodules, with the potential to expand the range of patients who can undergo surgery (156). NBNC-HCC has a higher incidence rate in developed countries, which is related to the regional economy, diet, and medical level. With the popularization of vaccination and the development of the economy and medical treatment, the infection rates of HBV and HCV in some countries and regions have gradually decreased. At the same time, greater consideration should be given to NBNC-HCC.

### 7.3 AI

With the development of artificial intelligence (AI), more and more AI technologies are being applied in medical and health services. For example, using omics data, machine learning (ML) and deep learning (DL) are used to predict metastasis (157). AI has been widely reported in the early diagnosis of HCC, such as the prediction model of HBV reverse transcriptase sequence combined with ML, establishing genome-wide interrogation of somatic copy number aberrations by ML as a non-invasive HCC detection, combining B-mode ultrasound with DL to identify AFP negative HCC (158–160). In terms of prognosis, artificial neural network model is helpful to preoperatively evaluate the HCC patients’ risk of liver failure and predict life quality after hepatectomy (161, 162). Based on DL of HCC pathological sections, new histological scoring methods can be established to predict tumor recurrence (163, 164). The combination of ML and CT radiomics can also predict the recurrence and MVI in solitary HCC (165, 166). Besides HBV-related HCC, ML can also be used to predict the risk of HCV-related HCC, assess DAA treatment for HCV, screen NAFLD in the general population, and select appropriate patients undergoing hepatectomy to replace liver transplantation (167–170).

## 8 Discussion

Primary liver cancer is one of the most common cancers in the world, with approximately 75% - 85% of cases being HCCs (1). Cases of HCC caused by HBV infection are common in HBV-endemic areas, including East Asia, Southeast Asia, and countries in the south of the Sahara Desert, most of which are low- and middle-income countries. The current situation is closely related to the living habits of local people, regional economy and health levels. As a remarkable example, China, a region with a high prevalence of HBV and a high incidence of HCC, has benefited from the popularization of HBV vaccination since the 1990s and the HBV infection rate among millennials declined significantly. For the foreseeable future, it is expected that patients with HBV-related HCC in similar countries and regions will decrease with vaccine popularity, which is in line with global health sector strategies on viral hepatitis. In addition to early prevention, early diagnosis and treatment of HBV-related HCC are vital. Hepatectomy is the primary treatment method for early- and middle-stage HCC. Meanwhile, many clinical trials are conducted to convert resectable tumors from advanced HCC. Therefore, it is essential to assess the prognosis of patients with HBV-related HCC who are scheduled for surgical treatment during the perioperative period in order to establish correct disease expectations and develop personalized therapy.

Clinical applications tend to favor predictors that are simple, fast, and non-invasive. Indicators commonly used to predict the prognosis of HBV-related HCC, such as AFP and HBV DNA level in serum, imaging results by US and CT. Unfortunately, to our best knowledge, most predictors in various studies do not have standardized thresholds which may be related to individual characteristics, dynamic changes in disease, or perioperative differences. This situation implies that it is impossible to establish a simple prediction model for HBV-related HCC after hepatectomy. This review covers the research on the preoperative and postoperative predictors of HBV-related HCC after hepatectomy over the past decade. Researchers have studied more extensive numbers and types of predictors, including unconventional blood test factors, genetic and pathological predictors, and other predictors. Factors such as population differences, regional medical level differences, dynamic changes of various indicators, and mass medical data on the disease process are obstacles to accurately predicting the prognosis of HBV-related liver cancer after hepatectomy.

A variety of HCC prognostic systems have been proposed internationally, focusing on liver function, tumor size, lymph node metastasis, and distant metastasis. Commonly used in clinical practice include Barcelona Clinic Liver Cancer (BCLC) staging, United Network for Organ Sharing (UNOS) tumor node metastases (TNM) staging, Hong Kong Liver Cancer (HKLC) staging, and American Joint Committee on Cancer (AJCC) TNM staging. These evaluation standards have been applicable to a large number of clinical practices. These staging systems are significantly correlated with prognosis. However, prognosis is also influenced by other factors such as overall health status of the patient, pathological characteristics of tumors, and serum tumor marker levels. These factors further affect prognosis assessment and follow-up treatment choices in HCC patients. Only through a thorough evaluation can the prognosis of patients be accurately predicted and realized precise medicine. To sum up, it is important to study other factors that can influence prognosis due to the following reasons: 1. Limitations of staging: While staging systems provide valuable information about the tumor, they may not encompass all factors that can impact prognosis. Other factors such as overall health condition of the patient, tumor differentiation, pathological characteristics of tumors, and tumor marker levels, can also influence the prognosis. 2. Individual variations: HCC is a highly heterogeneous tumor, and patients can exhibit significant differences in disease characteristics and biological behavior. Therefore, relying solely on staging systems may not accurately predict the prognosis for each patient. Studying other factors can help better understand the specific circumstances of individual patients and provide a more accurate assessment of their prognosis. 3. Diverse treatment strategies: HCC can be treated through various methods including surgical resection, liver transplantation, radiofrequency ablation, radiation therapy, chemotherapy, etc. The impact of different treatment modalities on prognosis may vary depending on the patient’s disease characteristics and staging. Hence, studying other factors can help determine the optimal treatment strategy for achieving a better prognosis. 4. Personalized prognosis: HCC is a highly personalized disease, and the prognosis can differ for each patient. By studying other factors that influence prognosis, it becomes possible to provide patients with more personalized treatment and care, thereby improving their survival rates and quality of life.

In our opinions, single or several predictive factors or a fixed model in the past do not meet the trend of personalized and precise medicine in the future. We want to establish a prediction system beyond traditional staging-related factors. Incorporating more dimensional parameters for personalized prediction is a significant foundation for achieving personalized and precise medicine. The development of AI technology and its gradual application in the medical field may provide a solution to the above issues. AI has been used to predict metastasis and tumor recurrence, as well as evaluate the HCC patients’ risk of liver failure and life quality after hepatectomy (157, 161–164). It is expected that AI will become more widely used in the medical field and that the difficulties that hinder the establishment of the prediction model of prognosis in the perioperative period will be overcome within a few years. Our research aims to incorporate factors other than commonly used tumor status and liver function, such as HBV DNA, HBsAg, multiple tumor pathological features, imaging features, etc., and utilize big biological data and AI to make more accurate predictions and provide follow-up treatment plans. In the foreseeable future, more extensive and in-depth cooperation among medical institutions, regions, and even countries will be needed.

## 9 Conclusion and perspectives

**Figure 2:**
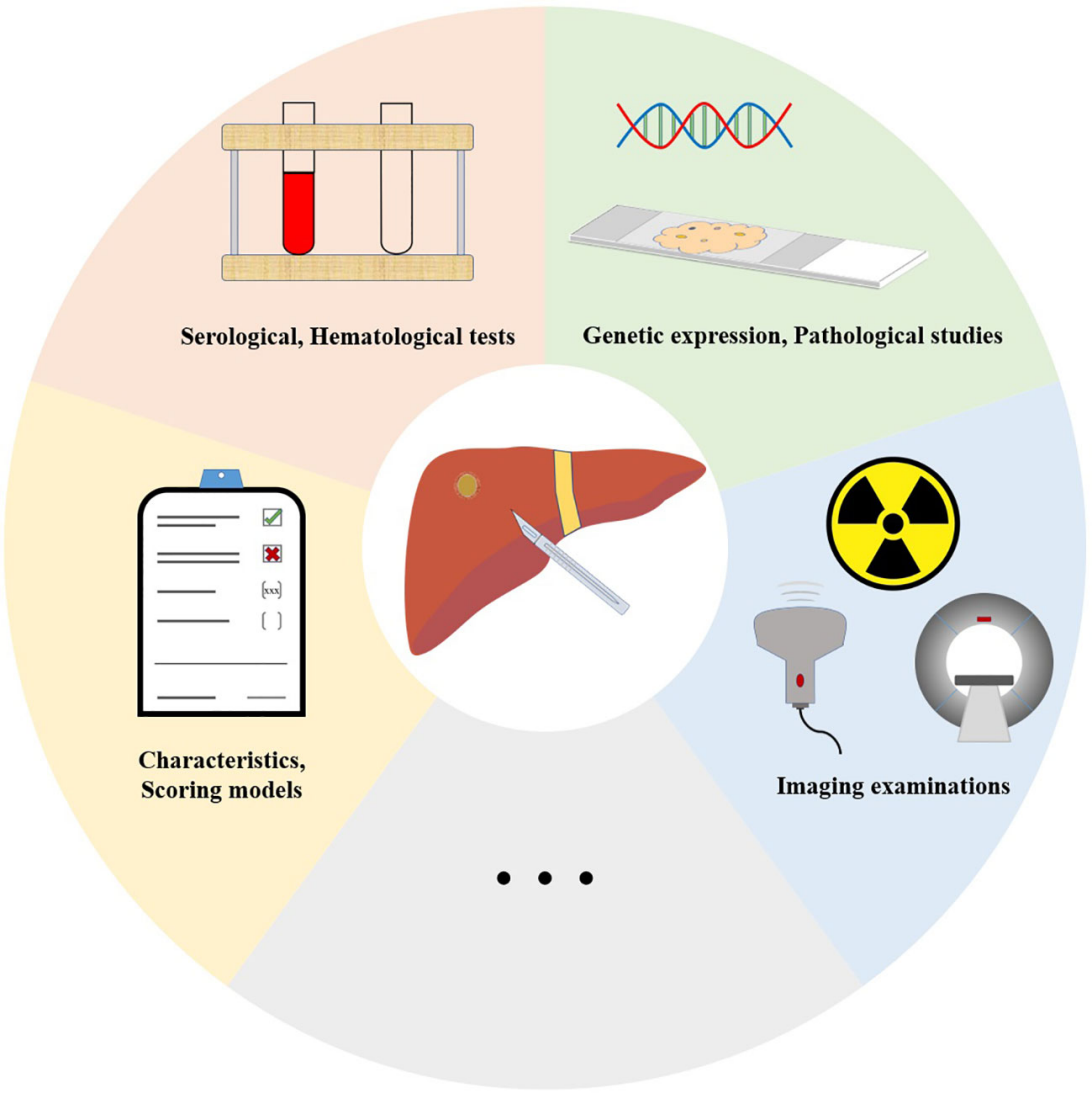
Various preoperative and postoperative predictors for the prognosis of HBV-related HCC.

To our best knowledge, the relevant studies in a decade adopted in this review are summarized in **Tables 1**–**4**, and **Figure 2**. As we all know, there are plenty of preoperative and postoperative factors that can be served as prognostic predictors, but some predictors have no standardized threshold. This situation may be related to the endemic distribution characteristics of HBV-related HCC, which are affected by biological characteristics (e.g., race, HBV genotype), as well as economic, medical (e.g., vaccination, linkage to care), and other factors. With the development and application of big biological data and AI, we expect that future medicine can conduct big data research in combination with various preoperative and postoperative predictors of HBV-related HCC, reasonably use AI to make more accurate prognosis prediction, establish correct disease expectations, and guide precise medicine. Achieving this goal requires a wide range of regions, a large number of medical institutions, and medical personnel to work together, which is a small step toward promoting human health.

## Author contributions

Conception and design: ZH, DT. Collection, analysis and writing: ZH. Review and modification: DT. All authors contributed to the article and approved the submitted version.

## Glossary

**Table:**

AFP	alpha-fetoprotein
AI	Artificial Intelligence
ALBI	albumin-bilirubin
ALP	alkaline phosphatase
ALT	alanine aminotransferase
APRI	aspartate aminotransferase-to-platelet ratio index
AUC	area under receiver operating characteristic curve
BCLC	Barcelona Clinic Liver Cancer
Bregs	regulatory B cells
CA19-9	carbohydrate antigen 19-9
CCA	cholangiocarcinoma
cccDNA	covalently closed circular DNA
CCI	comprehensive complication index
cfDNA	cell-free DNA
CHB	chronic hepatitis B
CK19	cytokeratin 19
COI	cut-off index
COUNT	controlling nutritional status
CRP	C-reactive protein
CSCs	cancer stem cells
DAAs	direct acting antiviral agents
DCP	des-γ-carboxy prothrombin
DFS	disease-free survival
DL	deep learning
ELISA	enzyme-linked immunosorbent assay
ER	early recurrence (within 2 years)
ES	embryonic stem
GGT	gamma-glutamyl transpeptidase
GPR	gamma-glutamyl transpeptidase to platelet ratio
HBeAg	hepatitis B e antigen
HBsAg	hepatitis B surface antigen
HBV	hepatitis B virus
HCC	hepatocellular carcinoma
HCV	hepatitis C virus
HCVAb	hepatitis C antibody
HPSE	heparinase
ICG-R15	indocyanine green retention rate at 15 min
IHC	immunohistochemistry
IL	interleukin
IVIM	intravoxel incoherent motion
LDH	lactate dehydrogenase
LMR	lymphocyte-to-monocyte ratio
lncRNA	long non-coding RNA
LR	late recurrence (over 2 years)
LSM	liver stiffness measurement
MACC1	metastasis-associated in colon cancer 1
ML	machine learning
MRE	magnetic resonance elastography
MRI	magnetic resonance imaging
MVI	microvascular invasion
NAFLD	non−alcoholic fatty liver disease
NBNC	non-HBV non-HCV
NLR	neutrophil-to-lymphocyte ratio
ORF	open reading frames
OS	overall survival
PET/CT	positron emission tomography/computed tomography
PLR	platelet-to-lymphocyte ratio
PNI	Prognostic Nutritional Index
POSTN	periostin
rcDNA	relaxed circular DNA
RFA	radiofrequency ablation
RFS	recurrence-free survival
sFLR	standardized future liver remnant
ST	splenic thickness
SVR	sustained virological response
T2DM	type 2 diabetes mellitus
TACE	transcatheter arterial chemoembolization
TE	transient elastography
TFS	tumor-free survival
Tregs	regulatory T cells
TS	tumor stiffness
US	ultrasound
WFA+-M2BP	Wisteria floribunda agglutinin-positive Mac-2-binding protein
γ-GT	γ-glutamyl transferase
18F-FDG	F-18-fluoro-2-deoxy-glucose
